# Cerebral Accumulation of Gadolinium (Gd^3+^) and Related Cellular Stress Pathways in Rat Brain Tissue

**DOI:** 10.3390/tomography12030037

**Published:** 2026-03-05

**Authors:** Göksel Tuzcu, Burak Çildağ, Songül Çildağ, Çiğdem Yenisey, Zahir Kızılay

**Affiliations:** 1 Department of Radiology, Faculty of Medicine, Aydin Adnan Menderes University, 09100 Aydin, Türkiye; mbcildag@yahoo.com; 2 Department of Immunology and Allergy, Faculty of Medicine, Aydin Adnan Menderes University, 09100 Aydin, Türkiye; songulcildag@yahoo.com; 3 Department of Biochemistry, Faculty of Medicine, Aydin Adnan Menderes University, 09100 Aydin, Türkiye; cyen2006@yahoo.com; 4 Department of Neurosurgery, Faculty of Medicine, Aydin Adnan Menderes University, 09100 Aydin, Türkiye; zahir.kizilay@adu.edu.tr

**Keywords:** gadolinium-based contrast agents, cerebral gadolinium accumulation, unfolded protein response, oxidative stress, rat model

## Abstract

Background/Objectives: This study aimed to compare in vivo cerebral gadolinium (Gd^3+^) accumulation, associated unfolded protein response (UPR), and oxidative stress parameters in rats after exposure to gadolinium-based contrast agents (GBCAs). Methods: This study was designed as a controlled, experimental animal study to evaluate the accumulation of Gd^3+^ in the basal ganglia of rats following the administration of 0.6 mmol/kg gadopentetate dimeglumine (linear) and gadoterate meglumine (macrocyclic). Male Sprague–Dawley rats were exposed to the contrast agents for 24 and 72 h, and then the basal ganglia tissues were collected postmortem. The tissue levels of Gd^3+^ accumulation, activating transcription factor-6 (ATF6), inositol-requiring enzyme-1 (IRE-1), protein kinase RNA-like endoplasmic reticulum kinase (PERK), damage-inducible transcript-3 (DDIT3), total antioxidant status (TAS), and total oxidant status (TOS) were determined. Results: Linear GBCA-treated rats had persistent Gd^3+^ levels over time, whereas a significant reduction from 24 to 72 h was observed in macrocyclic GBCA-treated rats (*p* < 0.001). PERK, DDIT3, and ATF6 expressions were significantly elevated after linear GBCA exposure (*p* < 0.05), while no significant increase was observed in the macrocyclic GBCA-treated group. However, IRE-1, TAS, and TOS levels were not significantly different in either group. Conclusions: Linear and macrocyclic GBCAs demonstrated distinct patterns of cerebral Gd^3+^ accumulation and UPR levels in rats. Accordingly, GBCA administration should be reserved for instances where it is necessary, such as when contrast enhancement is clinically required.

## Simple Summary

Cerebral gadolinium (Gd^3+^) accumulation, unfolded protein response level, and oxidative stress were studied in rat basal ganglia after exposure to gadopentetate dimeglumine (linear) and gadoterate meglumine (macrocyclic). Both agents resulted in Gd^3+^ accumulation in cerebral tissue at 24 and 72 h; however, it remained stable with the linear agent, while a time-dependent decline was observed with the macrocyclic agent. Elevated levels of PERK, DDIT3, and ATF6 were observed with the linear agent, in contrast to the macrocyclic agent, which did not reveal a significant stress response. In addition, IRE-1, TAS, and TOS remained significantly unchanged. Overall, the two agents showed different accumulation properties and cellular stress profile patterns, supporting the preferential use of macrocyclic GBCAs.

## 1. Introduction

Gadolinium (Gd^3+^), a member of the lanthanide family, is widely used in clinical practice as a contrast agent for magnetic resonance imaging. Gd^3+^-chelated derivatives have been used extensively in clinical imaging since 1988 and have a safety profile characterised by low rates of acute adverse reactions in the range of 0.01–2% [1]. Over the past decade, there has been a dramatic increase in human exposure to Gd and its derivatives. As human exposure to Gd^3+^-based contrast agents (GBCAs) increases, concern has emerged about tissue retention and their potential adverse effects. Autopsy-based studies have shown Gd^3+^ accumulation in human brain tissue after repeated administration of GBCAs, even in the absence of severe renal dysfunction or underlying intracranial pathology [2,3]. In addition, Gd^3+^ retention in the brain after repeated administration of intravenous GBCAs was also shown in MRI-based animal and human studies [4]. Increased signal intensity on unenhanced T1-weighted images of the dentate nucleus and globus pallidus has been linked to chelate structure, stability, and cumulative exposure to GBCAs [5,6]. In addition, exposure to linear or macrocyclic GBCAs was not associated with significant differences in motor or behavioural function in mice [7].

Metal ion accumulation within neural tissue is characterised by disruption of intracellular redox homeostasis and calcium signalling. This disturbance is associated with increased reactive oxygen species (ROS) generation and subsequent organelle dysfunction, particularly in mitochondria and the endoplasmic reticulum (ER), contributing to multiple pathologies [8]. Although free radicals are essential for normal cellular signalling and immune function, their excessive production disrupts redox homeostasis, leading to oxidative stress and organelle dysfunction [9]. ER stress is characterised by the accumulation of misfolded or unfolded proteins resulting from impaired protein homeostasis, including chaperone-mediated folding and ER-associated degradation.

Activation of the unfolded protein response (UPR) as a result of severe or persistent stress is associated with inflammatory signalling and cell death pathways [10]. ER-resident signalling molecules, including ATF6, IRE1, PERK, and BiP (GRP78), are essential components of the UPR in mammalian cells, with a central role in cellular adaptation to ER stress [11]. ATF6 serves as a transcriptional regulator of ER chaperone and protein-folding enzyme expression; IRE1 acts as a signal transducer mediating activation of adaptive and stress-response genes through mRNA splicing and downstream signalling; and PERK is a regulator of global protein synthesis, with attenuation of ER protein load and selective promotion of stress-response and cell-survival pathways.

GBCA exposure is associated with Gd^3+^ release and subsequent tissue deposition, with short- and long-term toxic effects reported in renal, hepatic, neural, and macrophage cells [12]. Inflammatory signalling, oxidative stress, apoptotic pathway activation, and ER stress are proposed mechanisms of Gd^3+^-induced cytotoxicity, supported by in vitro rat cortical neuron models characterised by oxidative injury-associated toxicity and activation of ER stress pathways [13,14]. The existing mechanistic evidence is largely derived from in vitro neuronal models and reports of in vivo cerebral accumulation. In addition, the associated ER stress responses remain poorly characterised. Therefore, this study set out to evaluate the accumulation of linear and macrocyclic GBCAs and to investigate the associated ER stress-related pathways and oxidative stress parameters in the rat brain basal ganglia.

## 2. Materials and Methods

### 2.1. Ethical Statement

The present study was approved by the Local Ethics Committee for Animal Experiments at Aydin Adnan Menderes University (approval no: 64583101; date: 17 January 2019).

This study was conducted and reported in accordance with the ARRIVE 2.0 guidelines, and the ARRIVE checklist was used as a guide for reporting the animal experiments.

### 2.2. Study Design and Subjects

A total of 40 male Sprague–Dawley rats (8–10 weeks old; body weight 250–300 g) were used in this comparative experimental animal study. The rats were housed under standard laboratory conditions (temperature 22 ± 2 °C, 12 h light/dark cycle) with ad libitum access to standard chow and water in the Laboratory Animal Centre of the Adnan Menderes University Faculty of Medicine Experimental Animals Unit. Animals were group-housed in standard cages and provided with routine environmental enrichment. All animals were allowed to acclimatise to the laboratory environment prior to the initiation of experimental procedures.

Gadopentetate dimeglumine (Magnevist^®^, Bayer Schering Pharma, Berlin, Germany), a linear Gd^3+^-based contrast agent, and gadoterate meglumine (Dotarem^®^, Guerbet, Villepinte, France), a macrocyclic Gd^3+^-based contrast agent, were used in the experiment. Rats were randomly allocated into five experimental groups (*n* = 8 per group): control (saline, 24 h), linear—24 h, linear—72 h, macrocyclic—24 h, and macrocyclic—72 h. Contrast agents were administered via tail vein injection at a dose of 0.6 mmol/kg. Each rat received the GBCAs only once at the beginning. Although this dose exceeds those used in clinical practice, it has been widely applied in experimental rodent models to achieve measurable cerebral Gd^3+^ accumulation for quantitative and mechanistic analyses. Control animals received an equivalent volume of saline via the same route.

All experimental procedures were performed by trained personnel, and appropriate anaesthesia was used during invasive procedures to minimise pain, suffering, and distress. Animals were monitored daily for general health status and signs of discomfort throughout the study period.

### 2.3. Cerebral Accumulation of Gadolinium

The rats were sacrificed after 24 or 72 h to determine baseline cerebral Gd^3+^ levels. At each predefined time point (24 h and 72 h), the animals were deeply anaesthetised and sacrificed by decapitation. Brain tissues were rapidly excised and stored at −80 °C until analysis. For Gd^3+^ quantification, approximately 1 g of postmortem basal ganglia tissue was digested in concentrated nitric acid using a closed Teflon digestion system, boiled for 15 min, and diluted to a final volume of 50 mL. Gd^3+^ concentrations were measured via inductively coupled plasma mass spectrometry (ICP-MS; Agilent 7700 Series, Santa Clara, CA, USA) using a standard calibration curve (0.1–20 µg/g) prepared from certified Gd^3+^ standards. Results were expressed in parts per million (ppm).

### 2.4. Determination of Endoplasmic Reticulum Stress Response

ER stress response was evaluated by measuring the brain tissue levels of inositol-requiring enzyme-1 (IRE-1), protein kinase RNA-like ER kinase (PERK), activating transcription factor-6 (ATF6), and DNA damage-inducible transcript-3 (DDIT3/CHOP) at each sampling time point. Brain tissues collected at 24 h and 72 h after the administration of the contrast agent or saline were homogenised in RIPA buffer (Thermo Fisher Scientific, Waltham, MA, USA) supplemented with protease inhibitors. Homogenates were centrifuged at 12,000× *g* for 20 min at 4 °C, and the resulting supernatants were used for analysis. ER stress marker concentrations were determined using commercially available enzyme-linked immunosorbent assay (ELISA, Elabscience, Wuhan, China) kits according to the manufacturer’s instructions. All assays were performed in duplicate, with absorbance measured at 450 nm using a microplate reader (BioTek, Minneapolis, MN, USA). Concentrations were calculated from standard curves and normalised to protein content, expressed as ng/mg protein.

The 24-h time point was selected to reflect early ER stress signalling associated with acute Gd^3+^ exposure, whereas the 72-h time point was chosen to evaluate sustained or delayed ER stress responses potentially related to cerebral Gd^3+^ retention.

### 2.5. Determination of Oxidative Stress Parameters

To determine the effect of Gd^3+^ on the pro-oxidant/oxidant balance and assess oxidative stress, total oxidant status (TOS) and total antioxidant status (TAS) levels were measured in brain tissues obtained at 24 h and 72 h following administration of the contrast agent or saline. TAS and TOS analyses were performed on the same brain tissue homogenates used for ER stress evaluation, using commercially available ELISA kits in accordance with the manufacturers’ protocols. Measurements were conducted in duplicate, and concentrations were calculated using standard calibration curves. Oxidative stress parameters were analysed in parallel with ER stress markers at both time points to assess the temporal relationship between Gd^3+^-induced oxidative imbalance and ER stress activation.

### 2.6. Statistical Analysis

The statistical analysis of the acquired data was conducted utilising the GraphPad Prism version 9.0 (GraphPad Software, San Diego, CA, USA). Data distribution was assessed for normality using the Shapiro–Wilk test. As all variables demonstrated normal distribution, results were expressed as mean ± standard deviation (SD). The measurements among the five groups were compared using one-way analysis of variance (ANOVA), and post hoc multiple comparisons were conducted using Tukey’s honestly significant difference (HSD) test to identify intergroup differences.

Two-way correlation tests were employed to ascertain the presence of positive or negative correlations among the groups based on the doses of linear and macrocyclic agents (*p* < 0.05). The groups were assessed internally using a table presenting the mean ± standard deviation. Each signal molecule’s measurements in the groups were depicted separately on the charts. Statistical analyses were performed using appropriate statistical software.

## 3. Results

The present study evaluated cerebral accumulation of Gd^3+^, UPR markers, and oxidative stress parameters in a rat model.

### 3.1. Gadolinium Accumulation in Brain Tissue

Cerebral Gd^3+^ concentrations differed significantly among groups (Table 1). In the linear-treated rats, Gd^3+^ levels were significantly higher than in controls at both 24 h (87.11 ± 4.03 ppm vs. 5.1 ± 3.02 ppm, *p* < 0.0001) and 72 h (82.7 ± 2.02 ppm, *p* < 0.0001). The change in Gd^3+^ levels at 72 h compared with 24 h in the linear agent group was not statistically significant (*p* > 0.05). A general examination of the rats was performed, and no abnormalities or clinical signs of illness were detected. Additionally, the animals exhibited no observable neurological changes.

In the macrocyclic agent-treated groups, Gd^3+^ accumulation was also detectable at 24 h and 72 (79.13 ± 2.03 ppm vs. 5.1 ± 3.02 ppm, *p* < 0.0001; 54.94 ± 3.05 ppm vs. 5.1 ± 3.02 ppm, *p* < 0.001). A significant decrease was noted between 24 and 72 h (79.13 ± 2.03 vs. 54.94 ± 3.05; *n* = eight per group; *p* < 0.001).

### 3.2. Unfolded Protein Response and Oxidative Stress Parameters

**Figure 1:**
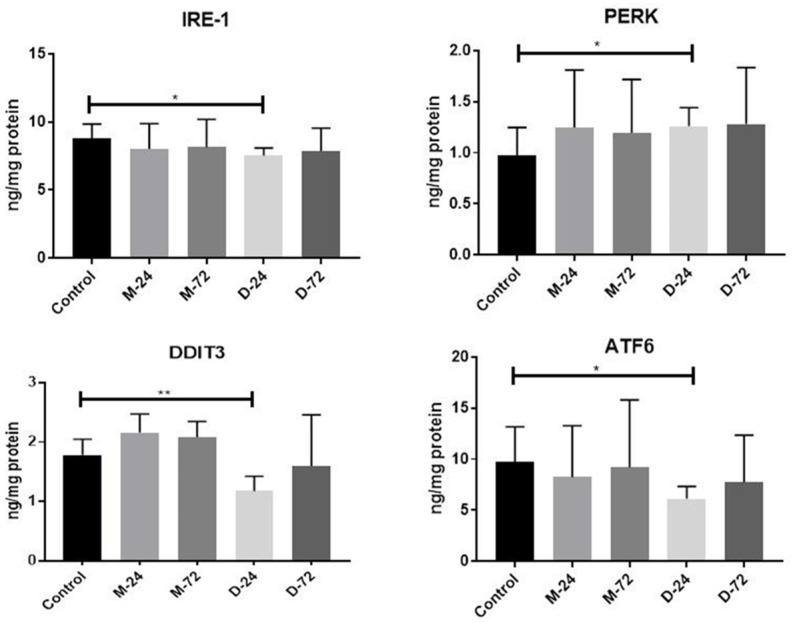
Graphical representation of UPR protein levels in Magnevist- and Dotarem-treated rats compared with the control group (* *p* < 0.05; ** *p* < 0.01). M-24, Magnevist at 24 h; M-72, Magnevist at 72 h; D-24, Dotarem at 24 h; D-72, Dotarem at 72 h; IRE-1, inositol-requiring enzyme 1; PERK, protein kinase RNA-like endoplasmic reticulum kinase; DDIT3, DNA damage-inducible transcript 3 (CHOP); ATF6, activating transcription factor 6.

**Figure 2:**
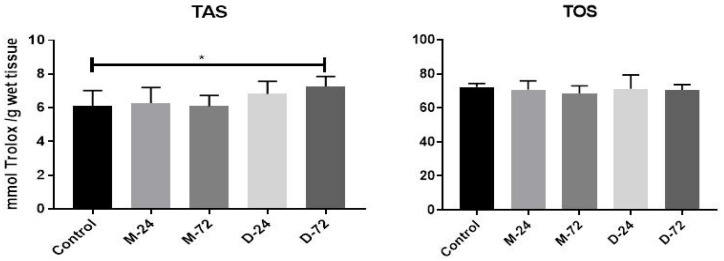
Graphical representation of TAS and TOS levels in Magnevist- and Dotarem-treated rats compared with the control group (* *p* < 0.05). M-24, Magnevist at 24 h; M-72, Magnevist at 72 h; D-24, Dotarem at 24 h; D-72, Dotarem at 72 h; TAS, total antioxidant status; TOS, total oxidant status.

The results of the quantitative analysis of ER stress-related UPR markers are summarised in Figure 1. IRE-1 expression did not differ significantly among the study groups at either time point (*p* > 0.05). PERK protein levels were significantly increased in the linear GBCA-treated groups at both 24 h and 72 h compared with the controls (*p* < 0.01 for both), whereas no significant change was observed in the macrocyclic agent-treated groups (*p* > 0.05). Similarly, DDIT3 and ATF6 expressions were significantly elevated following administration of the linear agent at 24 h and 72 h relative to the controls (*p* < 0.05 for both). In contrast, no significant changes in DDIT3 or ATF6 expression were observed in macrocyclic agent-treated animals at either time point (*p* > 0.05), nor were any significant differences observed between the groups in TAS and TOS measurements (Figure 2). Overall graphical comparisons of UPR marker expression in linear- and macrocyclic agent-treated rats relative to controls are presented in Figure 1.

## 4. Discussion

The paramagnetic property of Gd^3+^ is the key to its application in medical diagnosis and treatment monitoring, with GBCAs being used in approximately 25% of MRI examinations. As free Gd^3+^ is toxic, it is administered in clinical practice as linear or macrocyclic chelated compounds, which reduce toxicity and facilitate elimination [15]. The accumulation of contrast agents in the brain, even after standard use, has been well documented in both human and animal studies, raising concerns about long-term or repeated use [16,17]. In this study, we examined the accumulation of Gd^3+^ in rat brain tissue after linear and macrocyclic GBCA administration and the changes in ER stress-related markers and antioxidant levels. A single intravenous dose of both agents resulted in detectable Gd^3+^ accumulation in the basal ganglia, with higher accumulation observed in the linear agent. The retention decreased by 72 h for both agents, but the decline was more pronounced for the macrocyclic agent. These findings were in accordance with a number of studies reporting that linear GBCAs cause higher brain accumulation than macrocyclic agents, although macrocyclic GBCAs can also result in detectable retention, particularly after repeated applications [18,19,20,21]. Similarly, a small but statistically significant, dose-dependent T1-weighted signal increase in the dentate nucleus was detected after repeated macrocyclic GBCA administrations, with visible enhancement in patients receiving 37 to 44 cumulative doses [22]. Brain gadolinium retention occurs cumulatively following repeated administrations and is generally asymptomatic, although potential cognitive effects remain a subject of debate. As our study was designed to investigate potential biochemical alterations through a mechanism-based approach and was conducted on animal subjects, we were unable to observe any clinical outcomes. Gadolinium deposition disease (GDD) manifests within hours to weeks post-exposure and is characterised by persistent systemic symptoms, including headache, musculoskeletal pain, fatigue, cognitive complaints, and sensory disturbances, without established objective findings. Conversely, acute hypersensitivity reactions arise within minutes to hours following GBCA administration, presenting with symptoms such as nausea, vomiting, rash or urticaria, and, in rare instances, anaphylactoid reactions [23].

Gd^3+^ compounds have toxic effects, including reduced motility, irreversible attachment to the growth surface, and cell death [24]. ROS-related toxicity of GBCAs was evidenced by mitochondrial membrane potential loss, chromatin condensation, altered Bcl-2/Bax expression, and DNA fragmentation in cell lines [25]. Gd^3+^ exposure has been shown to increase ROS production and induce key ER stress markers such as BiP/GRP78 and XBP1 [26]. In the present study, we evaluated early UPRs after single-dose exposure to GBCA in the rat brain. The most important finding was that the linear agent increased the expression of PERK, DDIT3, and ATF6, whereas the macrocyclic agent did not induce a significant change in ER stress-related markers. Differences observed between the linear and macrocyclic agents in our study are consistent with reports that linear GBCAs exhibit lower chelation stability and greater biological reactivity, leading to stronger cytotoxic and stress-related responses than macrocyclic agents [27]. Exposure of lymphocytes to different GBCAs did not produce a measurable increase in markers of DNA double-strand breaks; however, enhanced cytotoxicity was observed only in lymphocytes treated with the linear GBCAs [28]. Additionally, recent studies highlight that agent structure influences intracellular signalling, with linear agents showing a higher propensity to disrupt homeostasis and promote ER stress-related pathways [29]. Although generally unfavourable, GdCl_3_ can activate death receptor, mitochondrial, and ER stress pathways to induce apoptosis in osteosarcoma cells, suggesting a potential therapeutic role [30]. Our findings support prior evidence that GBCAs can induce neurotoxicity and apoptosis through activation of ER stress and unfolded protein response pathways [31]. This highlights the relative safety profile of Dotarem in clinical radiology practice. A review of 35 years of published data and pharmacovigilance monitoring found the macrocyclic agent to be a safe radiological agent for all age groups [32]. It seems possible that these results are due to differences in the chemical structure, stability, or kinetic behaviour of the contrast agents, which may influence their interaction with cellular stress pathways. Upregulated inflammation was reported in murine macrophages even at small levels of GBCAs [33].

In our study, stable TAS and TOS levels suggest that GBCAs do not consistently induce measurable systemic oxidative stress under experimental conditions. These data indicate that gadodiamide and gadoteric acid can induce hepatocellular necrosis and apoptosis despite minimal effects on total oxidant and antioxidant capacity [34]. Dihydroethidium staining in skin tissue was markedly increased in contrast-treated animals, consistent with enhanced reactive oxygen species production and elevated oxidative stress [35]. In addition, antioxidant and metal-chelation strategies have been shown to reduce GBCA toxicity. These approaches can improve renal outcomes, diminish NSF-related changes, and help prevent Gd^3+^ retention in tissues. The induction of apoptosis is mediated predominantly through mitochondrial-dependent pathways following Gd^3+^ exposure, with reactive oxygen species constituting key mechanistic mediators [12]. Exposure to Gd^3+^ reduces lymphocyte viability in a concentration- and time-dependent manner and increases DNA damage, apoptosis, and ROS production [36]. In addition, neuronal apoptosis can be induced by Gd^3+^ through mitochondrial dysfunction and oxidative stress, characterised by increased ROS production, ATP depletion, opening of mitochondrial membrane permeability, cytochrome c release, and caspase-3 activation [37]. Earlier work also demonstrated that Gd^3^-based compounds enhanced ROS, nitrate/nitrite, and PGE2 production, mitochondrial depolarisation, and inflammatory responses, with linear agents producing more pronounced effects than macrocyclic formulations [32]. Apoptosis of multiple cell types via mitochondrial injury, Ca^2+^ dysregulation, ER stress signalling, and death receptor pathways has been reported [12,29,30,36,37,38]. Overall, the data reinforce that Gd^3+^ can trigger oxidative and ER stress pathways, but the magnitude and persistence of these effects differ greatly between linear and macrocyclic GBCAs.

In Türkiye, it is estimated that approximately 10–12 million MRI examinations are performed annually. As many patients undergo repeated contrast-enhanced MRI studies during long-term follow-up, cumulative Gd^3+^ exposure has become an increasing concern. The FDA advises re-evaluating the necessity of repeated contrast-enhanced MRI examinations within the existing treatment protocols to reduce the potential for Gd^3+^ accumulation [39]. Healthcare professionals should also be aware of Gd^3+^ retention, particularly in patients such as those requiring multiple doses, pregnant women, children, and patients with inflammatory conditions. Repeated or closely scheduled contrast-enhanced MRI examinations should be minimised when possible, but clinically necessary GBCA-enhanced scans should not be avoided or delayed [40].

A limitation of this study was the relatively brief observation period, restricted to 24 and 72 h following GBCA exposure. While initial Gd^3+^ accumulation patterns rather than long-term retention were documented in our study, this does not represent long-term Gd retention in the brain. Even in individuals without significant renal impairment, the administration of GBCA results in the accumulation of gadolinium in the brain [2]. Previous research has evaluated deposition at later time points in the brains of healthy rats following multiple administrations of gadodiamide, revealing partial clearance over a 20-week period [41]. An extended observation period could provide further insights into the delayed clearance and potential persistence of Gd^3+^ in cerebral tissue. Therefore, future studies with longer observation intervals are necessary to better characterise the long-term deposition and clearance kinetics of gadolinium.

## 5. Conclusions

In conclusion, this study highlights the differential cerebral accumulation of Gd^3+^ and associated UPR following exposure to linear and macrocyclic GBCAs in rats. More persistent Gd^3+^ accumulation and significant upregulation of PERK, DDIT3, and ATF6 after linear GBCA administration contrast with the reduced accumulation and lack of significant UPR activation with macrocyclic GBCA. These findings underscore the importance of cautious and justified use of GBCAs in clinical practice to minimise potential neurotoxic effects related to Gd^3+^ retention and cellular stress responses. Further research is warranted to explore the long-term implications of these changes and to optimise contrast agent selection for patient safety.

## Figures and Tables

**Table 1:** Cerebral Gd^3+^ concentrations following administration of linear and macrocyclic GBCAs.

Group	*n*	Agents	Dose (mmol/kg)	Time	Route	Gd (ppm)
1	8	linear	0.6	24 h	Tail vein (IV)	87.11 ± 4.03 ^b^
2	8	linear	0.6	72 h	Tail vein (IV)	82.70 ± 2.02 ^c^
3	8	macrocyclic	0.6	24 h	Tail vein (IV)	79.13 ± 2.03 ^d^
4	8	macrocyclic	0.6	72 h	Tail vein (IV)	54.94 ± 3.05 ^e^
Control	8	Saline	—	24 h	Tail vein (IV)	5.1 ± 0.24 ^a^

IV, intravenous; Gd, gadolinium; ppm, parts per million. ^a^ significant difference compared to all other groups, *p* < 0.0001; ^b^ significant difference compared to control, group 3 and 4, *p* < 0.001; ^c^ significant difference compared to group 4 and control, *p* < 0.001; ^d^ significant difference compared to group 1, 4 and control, *p* < 0.001 ^e^ significant difference compared to all groups, *p* < 0.0001. Only statistically significances are noted. A *p* value < 0.05 was considered statistically significant.

## Data Availability

The data that support the findings of this study are available from the corresponding author upon reasonable request.

## Abbreviations

The following abbreviations are used in this manuscript:

GBCAs	Gadolinium-based contrast agents
Gd^3+^	Gadolinium ion
ROS	Reactive oxygen species
ER	Endoplasmic reticulum
UPR	Unfolded protein response
TAS	Total antioxidant status
TOS	Total oxidant status
PERK	Protein kinase RNA-like endoplasmic reticulum kinase
DDIT3	DNA damage-inducible transcript 3
ATF6	Activating transcription factor 6
CHOP	C/EBP homologous protein
